# The Made‐in‐Africa Evaluation framework: A decolonial approach to program evaluation

**DOI:** 10.1002/ajcp.70102

**Published:** 2026-08-18

**Authors:** Takatso Sibanda, Robin Lin Miller

**Affiliations:** ^1^ Department of Psychology Michigan State University East Lansing Michigan USA

**Keywords:** community psychology, decoloniality, Indigenous knowledge, international development, Made in Africa Evaluation, program evaluation

## Abstract

The Made in Africa Evaluation (MAE) framework is a decolonial approach to program evaluation developed by African evaluators over the past 14 years. MAE may be appropriate to community psychologists who practice program evaluation or conduct research in Africa, but little is known about its implementation. We conducted a scoping review to map the existing literature on MAE, examine its core practice tenets, explore how its principles are currently being operationalized, and identify gaps in the MAE literature. Eligible articles (*N* = 53) included primary conceptual papers, case studies and seminal conference proceedings that were published between 2012 and 2026. Findings indicated a strong focus on the use of Indigenous methods and Ubuntu philosophy, African ownership of evaluation processes and findings, and social transformation aligned with African values. Major gaps include few case studies of applying MAE and limited guidance on applying Ubuntu ethics in evaluation practice. MAE's values align closely with community psychology's core values of community self‐determination and collaborative action in pursuit of social justice. MAE holds promise to guide reflexive international evaluation practice within community psychology. Future research should explore how MAE is being applied and how it can be adapted across diverse practice contexts pertinent to community psychology.

## Highlights

- MAE provides community psychologists with a decolonial evaluation approach aligned with African values, cultural contexts knowledge systems, and lived experiences.
- Ubuntu encourages moral, reflexive evaluation through critical reflection on power, privilege, and ethics
- Community psychology and MAE can strengthen each other to advance socially just, community‐led, transformative evaluation.

In community psychology, program evaluation serves as both a form of inquiry and intervention anchored in social justice (Miller, 2017). Program evaluation is an essential approach to advancing justice and enacting social change because it assesses intervention relevance, efficacy, and effectiveness and provides evidence on whether planned interventions enhance quality of life (O'Connor, 2021). There are many community psychologists who are expert evaluators and emerging community psychologists must develop program evaluation expertise, which is a core area of competence in the discipline (Cook, 2014; Miller, 2017; Wolfe & Price, 2017; Wolfe, 2019). As evaluation scholars have long argued (Miller, 2017; Schröter et al., 2026; Scriven, 1991, 2008; Shadish, 1998; Smith, 2010), evaluation is not a method but a transdiscipline with a diverse array of theories of practice that vary in their rationales for deciding on critical matters such as how to select appropriate methods, ensure the use of findings, identify the values that should inform judgments about the merit or worth of the thing being evaluated, and involve those who have an interest in the program and its evaluation. Theories also address issues such as the extent to which social justice and change should be an objective of an evaluation. Community psychologists should be aware of the diversity of theories of practice in evaluation, including those that originate outside of the United States, to enable them to select evaluation approaches that are most aligned with the values of our field and with varying evaluation contexts.

Despite an orientation to social justice and community empowerment, evaluation practice in community psychology continues to be shaped by Western‐centric theories and assumptions about what counts as valid knowledge in evaluation, often adopting the dominant theories and practices that inadvertently reproduce the oppression we aim to transform (Malherbe et al., 2021; Reyes Cruz & Sonn, 2011; Rosario et al., 2024). This observation signals the importance of community psychologists becoming familiar with evaluation approaches that are better aligned with the diverse values, cultural contexts, knowledge systems, and lived experiences of the communities we serve. The need for familiarity with contextually grounded approaches is evident when considering that the field of psychology broadly has historically centered Western/Euro American ideas, and marginalized Indigenous and Global South perspectives (Bryant, 2024; Readsura Decolonial Editorial Collective & Malherbe et al., 2023; Readsura Decolonial Editorial Collective, 2022). This centering of Western epistemologies as the default standard shapes how community psychologists conceptualize and practice research and evaluation.

In response, current debates on decolonizing or transforming community psychology explore the imagination, illumination, and advancement of alternative, context‐specific forms of knowledge generation and practice that emerge from and are aligned to the diverse epistemologies, histories, and worldviews of communities (Bhatia et al., 2025; Readsura Decolonial Editorial Collective & Malherbe et al., 2023; Readsura Decolonial Editorial Collective, 2022). These debates build on longstanding traditions in Southern theory, critical community psychology, Indigenous and liberation psychologies, each of which emerged as critiques of the devaluing of non‐Western epistemologies and the privileging of a Euro‐American canon as the universal standard for the field (Bryant, 2024; Carolissen et al., 2017; Malherbe et al., 2021; Sonn et al., 2021, 2024). These traditions were borne out of the struggles of Indigenous, African, Black, Latin American, and other colonized or marginalized communities for self‐determination and epistemic agency. They resonate with context‐specific decolonial approaches to program evaluation that are emerging around the world (Chapman et al., 2026; Cho, 2024; Hassnain, 2023; Schröter et al., 2026).

## DECOLONIAL EVALUATION MOVEMENTS

Debates on the role of decolonial approaches to evaluation practice have occurred in parallel to those in community psychology. Established decolonial evaluation movements include Indigenous Evaluation (IE) (Bowman Lunaape/Mohican & Bremner Métis, 2024; Bowman, 2020, 2025; LaFrance & Nichols, 2008; LaFrance, 2004), Culturally Responsive Evaluation (CRE) (Hood et al., 2015; Hopson, 2009), Kaupapa Māori evaluation (Cram, 2016; Cram et al., 2018; Wehipeihana & McKegg, 2018; Wehipeihana, 2013, 2019), and Transformative evaluation (Mertens, 2009, 2023a, 2023b). These movements emerged in response to the need for evaluation that is responsive to the historical, social, and cultural contexts in which programs operate. They are rooted in diverse historical and geographic contexts, intellectual traditions, political struggles and aims (Lyons et al., 2017; Ndlovu‐Gatsheni, 2015) and have been shaped by the work and scholarship of diverse, formerly colonized, Indigenous peoples, and scholars across Africa, Aotearoa (New Zealand), Asia, Australia, Canada, Hawaii, Latin America, North America and Oceania (Chilisa, 2020; Cram et al., 2025). They emphasize that rather than impose external frameworks, evaluation must honor and emerge from community values, priorities, ways of living and knowing (Chilisa, 2020; Chouinard & Hopson, 2016; Cram et al., 2018; Dazzo, 2026; Keney & Reid, 2026; Montrosse‐Moorhead et al., 2024). In practice, they privilege inclusive, participatory, collaborative processes; community‐defined indicators of program success; and relational metrics that assess collective wellbeing (Cram et al., 2025). Like Southern theories, decolonial evaluation approaches contend with how the dominant knowledge systems that shape evaluation practice have historically marginalized Indigenous peoples and invalidated Indigenous knowledges (Bhatia et al., 2025; Fish & Gone, 2024).

## THE MADE IN AFRICA EVALUATION (MAE) MOVEMENT

One of the most recent decolonial movements in evaluation to emerge is the MAE movement (Chilisa, 2015, 2024; Ouédraogo et al., 2025). MAE arose over the past 14 years in response to the monumental and enduring epistemic, cultural, structural, and economic harm wrought by colonial dispossession in Africa (Pheko, 2024; Tuck & Yang, 2012). MAE situates itself within the broad international development context, in which a wide range of international development agencies and donors provide significant development assistance across Africa. Development aid to Africa often occurs under the banner of the Sustainable Development Goals with the intention to advance poverty alleviation and economic development through investments in infrastructure, health, sanitation, education, food security, and humanitarian relief (Tang & Bundhoo, 2017).

Development aid remains a highly contested topic in Africa, sparking fierce debate over its priorities, processes, implications, effectiveness, and long‐term impact (Acosta, 2017; Banks et al., 2015; Bhattacharya & Khan, 2020; Frehiwot, 2019; Lancaster, 1999; Moyo, 2009; Mpofu & Ndlovu‐Gatsheni, 2023). Critics of development aid point to the paradox that former colonizers recast themselves as benevolent donors and use aid to maintain influence over former colonies (Cho, 2024). These critiques also emphasize that African communities are not passive recipients of aid (Chilisa & Mertens, 2021; Chilisa, 2015; Masvaure & Motlanthe, 2022). They have long held their own indigenous, inter‐generational, relational counternarratives that predate and exceed donor‐driven aid models and drive innovations in sustainable natural resource management, agriculture, health and social welfare among others. The MAE approach is grounded in these community‐driven traditions, while in contrast, dominant models of development aid and its evaluation have excluded them (Boadu, 2025; Chilisa et al., 2016).

Rather than responding to community‐defined priorities, donors are viewed as channeling development aid to Africa in ways that advance their economic and political interests, while undermining African community agency by selecting and defining development problems, deciding on funding priorities, prescribing solutions, and establishing the metrics used in evaluating intervention success (Cho, 2024; Lyew et al., 2021). Consequently, despite decades of donor‐driven development programs, poverty and inequality persist across Africa (Moyo, 2009). But rather than addressing the deeper, structural roots of poverty or the barriers to transformative and sustainable change and examining why development interventions have been ineffective, donors prioritize evaluating programs to measure quantifiable impacts and account for resource use (Masvaure & Motlanthe, 2022; Ofir, 2013).

Within this landscape, development evaluation is driven by donors and powerful institutions in the global North that dictate the paradigms, approaches, theories, methods, priorities and conditions for evaluation (Chilisa & Mertens, 2021; Chilisa, 2015; Schröter et al., 2026). For donors, evaluation serves as a mechanism for demonstrating impact, guiding resource allocations, and enforcing accountability for development results (Chirau & Ramasobana, 2022; Cloete & Auriacombe, 2019; Masvaure & Motlanthe, 2022; Ofir, 2013). Hence, there are globally recognized evaluation standards and criteria which are “the core reference for evaluating international development and humanitarian projects, programs and policies” (Chirau & Ramasobana, 2022; Cloete, 2016; Dlakavu, 2023; Mapitsa et al., 2019; Murgatroyd & Feront, 2023; OECD/DAC Network on Development Evaluation, 2019, p. 2; Tirivanhu et al., 2018). Frameworks such as the OECD Development Assistance Committee (DAC) quality standards and criteria guide development evaluation in Africa and are widely used by donors, governments, implementing agencies, and evaluators. The frameworks are rooted in Western values and Western knowledge systems and have been criticized for the rigid measurement of quantifiable impacts without addressing the deeper issues of accountability to communities, and sustainable, transformative change (Chouinard & Hopson, 2016; Masvaure & Motlanthe, 2022; Ofir, 2013). Furthermore, they often apply externally defined metrics and standardized methodologies that are insufficient to capture the complexities of programs as they unfold in diverse sociocultural contexts in Africa (Mazigo, 2025).

## THE CURRENT STUDY

MAE confronts the incongruence between dominant development evaluation frameworks in Africa and African priorities, values and indigenous knowledge. MAE prioritizes African self‐determination and sustainable transformative change (Khumalo, 2022). MAE emerged in response to calls for evaluation approaches that align with the values, worldviews, priorities, and ways of being and knowing of African people (Chapman et al., 2026; Chilisa & Mertens, 2021; Chilisa, 2015, 2024; Murgatroyd & Feront, 2023; Ofir, 2018; Ouédraogo et al., 2025). In this sense, as an evaluation movement, MAE aligns deeply with the social justice and community self‐determination principles of community psychology. The synergy between the two reflects MAE's immense potential to enrich and draw from community psychology.

While MAE is gaining traction in some quarters of the evaluation community, little has been written about how the framework is applied and how it can contribute to the work of community psychologists practicing evaluation in Africa and other international contexts. We therefore conducted a scoping review (Arksey & O'Malley, 2005; Munn et al., 2018; Peters et al., 2024) to map out MAE's history and tenets while taking stock of its use and the gaps in existing MAE literature.

This review examines MAE as an Indigenous, decolonial evaluation framework to cultivate community psychologists' engagement with a broader range of evaluation theories developed outside the United States. It contributes to a synthesis of MAE's principles and illustrates how they are being applied in practice to inform community psychologists working in Africa and other international contexts that are like Africa in history, culture, and values. The African development context is central to this review because the continent receives substantial donor support, which is often evaluated through dominant, donor‐centric frameworks that prioritize quantifiable outcomes over the relational, contextual, and transformative dimensions of change valued by African communities (Ayele et al., 2025; Murgatroyd & Feront, 2023; Ofir, 2013). As community psychologists and scholars whose lives and work are deeply tied to the continent, we are cognizant of how evaluation practice in Africa can benefit from frameworks that honor African values, ways of knowing, and priorities, and aim to advance wider engagement with such approaches among community psychologists. Our scholarship and evaluation practices are grounded in Ubuntu and oriented toward transformative, sustainable change that uplifts African communities. These commitments shape how we approached this review and why we believe that community psychologists working in Africa, and other international contexts shaped by similar dynamics, stand to benefit from deeper engagement with decolonial evaluation approaches such as MAE.

## METHOD

We draw on the methodological guidelines articulated by Arksey and O'Malley (2005), Levac et al. (2010), and Peters et al. (2024) to identify the key scholarly contributions that have shaped the conceptual, methodological, and ethical contours of the MAE framework.

### Identification of literature

#### Manual search of leading MAE scholars

We used a multi‐phased, iterative literature search strategy that began with a manual search for works by leading MAE scholars ‐ Bagele Chilisa, Sulley Gariba, and Zenda Ofir ‐ on Google Scholar, PsycINFO, and ProQuest. Searches were conducted during July of 2025 and repeated in July of 2026. We prioritized starting with their writings because they are recognized thought leaders and architects of the MAE approach. Our initial scan yielded both peer‐reviewed and gray literature, including a blog post authored by Ofir (see Table 1).

**Table 1:** Key works by foundational MAE scholars.

Author	Key contributions
Bagele Chilisa	6 MAE‐focused papers and 2 book chapters
Sulley Gariba	1 development evaluation paper
Zenda Ofir	1 MAE‐focused blog post and 5 development evaluation papers

#### Targeted author and citation mining

Given Chilisa's extensive contributions to MAE, we subsequently conducted a targeted Google Scholar, ProQuest, and PsycINFO search of her MAE publications between 2012 and 2026, which is the timeframe that reflects the emergence of MAE scholarship. One landmark paper cited 161 times served as the anchor for citation mining, which surfaced other papers in the growing body of MAE literature.

#### Keyword‐Based database search

To broaden the search, we developed a set of iterative search terms informed by preliminary reading. Keywords included: “Made in Africa Evaluation,” “Made in Africa AND Evaluation,” “Africa AND Evaluation,” and “Africa AND Program Evaluation.” These terms were applied across Google Scholar, ProQuest, and PsycINFO, targeting peer‐reviewed English language articles published between 2012 and 2026. This strategy ensured an inclusive mapping of writing in the field, surfacing literature that may not have explicitly cited Chilisa but engaged substantively with the emergent MAE framework.

#### Gray literature and journal review

Recognizing the importance of gray literature in MAE's development, we manually searched African Evaluation Association (AfrEA) documents, conference proceedings, and *The African Journal of Evaluation*, which is the flagship regional journal on evaluation in Africa and which featured a special issue on MAE in 2022.

#### Dissertations and book chapters

Finally, we conducted a manual search of dissertations and book chapters to identify methodological and practice‐based MAE writing, including case studies.

### Study selection

After removing duplicates, we conducted a two‐stage screening process. First, abstracts were reviewed using the inclusion and exclusion criteria in Table 2.

**Table 2:** Inclusion and exclusion criteria for study selection.

Criterion	Inclusion	Exclusion
Language	English‐language publications or those translated into English	Publications in languages other than English
Date range	Published between 2012 and 2026, reflecting the emergence of MAE scholarship	Published before 2012 or outside the defined timeframe
Context	Studies situated in African evaluation contexts	Studies focused broadly on evaluation outside of the African program evaluation discourse
Conceptual focus	Substantive engagement with MAE's conceptual, methodological, or ethical, and practical dimensions	MAE mentioned without substantive engagement
Publication type	Peer‐reviewed articles, book chapters, dissertations, AfrEA documents, conference proceedings	Non‐peer‐reviewed documents such as blog posts, working papers, reports authored by individuals other than core MAE foundational scholars or not officially published by AfrEA

Inclusion was based on our judgment of an article's relevance to the review's aims. We included studies if they engaged substantively with the conceptual, methodological, ethical, or practical dimensions of MAE. In the second stage, we screened the full text to confirm eligibility and alignment with our review's guiding questions. As previously noted, we selectively included gray literature such as proceedings of a seminal AfrEA conference in 2012, and evaluation guidelines developed by AfrEA, as these provided unique insight into MAE's developmental trajectory and tenets.

### PRISMA‐ScR flow summary

**Figure 1:**
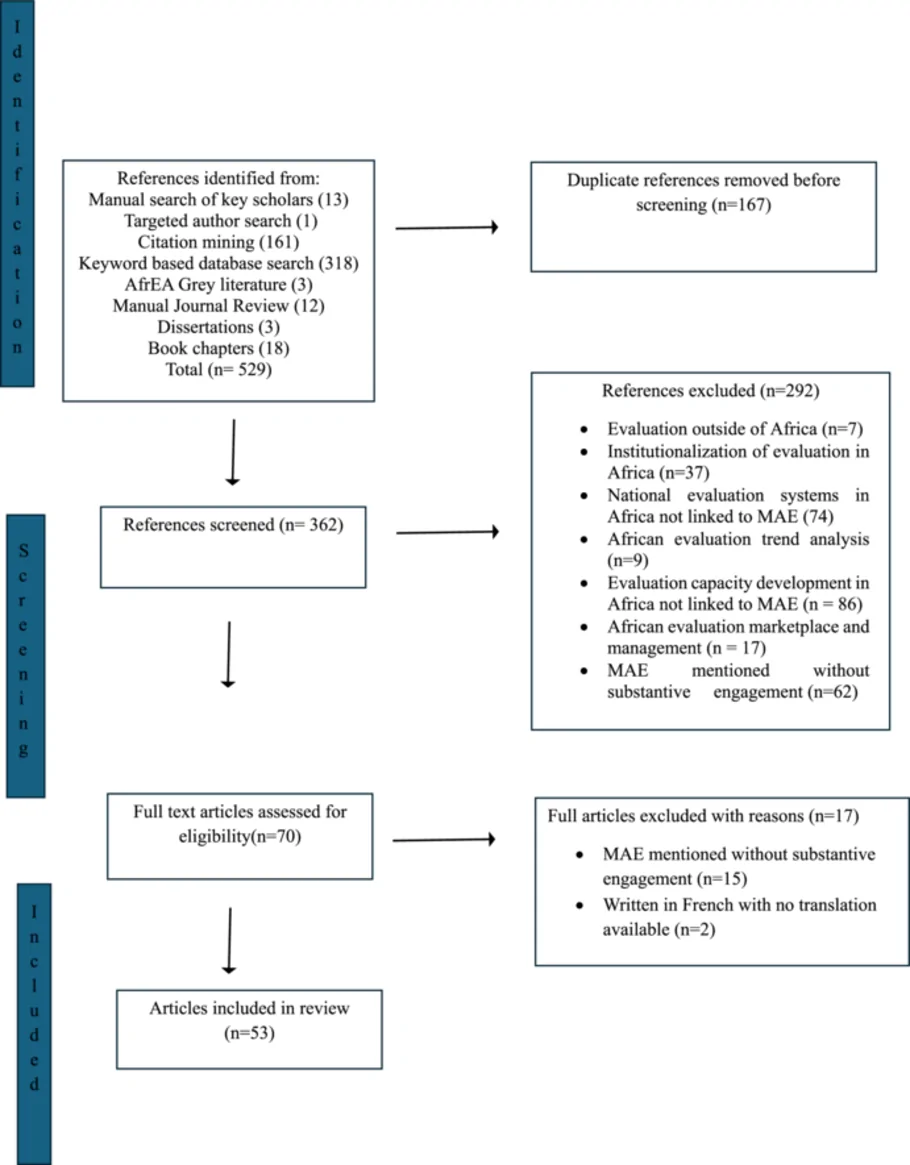
PRISMA flow diagram for study selection.

The study selection process is reported in the PRISMA‐ScR flow diagram (Figure 1) in line with the PRISMA‐ScR guidelines outlined by Tricco et al. (2018). The diagram illustrates the process of literature identification, screening, eligibility assessment, and inclusion across search phases. After removing duplicates and screening for potential inclusion, 53 documents were selected for data extraction and synthesis.

### Charting (extracting the data)

We reviewed each article and created a tabulated summary capturing key details, including the title, author(s), publication year, study purpose, and the specific MAE tenets addressed, such as historical development, definitions, theoretical foundations, and recommended methodologies.

### Collating, summarizing, and reporting the results

Next, we synthesized the extracted data by mapping studies according to the specific MAE tenets they reflected. Table 3 presents the distribution of papers across thematic areas, along with the number of scholars contributing to each area. Some papers offered expansive engagement across multiple tenets, appearing in more than one category, while others were more narrowly focused. This mapping revealed both the richness of conceptual contributions and a noticeable gap in the literature on the implementation of MAE in development programming contexts.

**Table 3:** Distribution of papers across MAE thematic areas.

Thematic area	Representative literature	Papers (*n*)
History	African Evaluation Association, (2007, 2013); Chilisa, (2015, 2024); Chilisa et al. (2025); Cloete (2016); Murgatroyd and Feront (2023); Frehiwot (2019); Keney and Reid (2026); Khumalo (2022); Masvaure and Motlanthe (2022); Mbava (2019); Mbava and Chapman (2020); Ofir (2018); Omosa (2019); Sefa‐Nyarko et al. (2024); Tirivanhu (2022); Tirivanhu et al. (2018)	17
Definition	African Evaluation Association (2021); Badjo (2022); Chilisa (2015, 2024); Chilisa and Mertens (2021); Omosa (2019); Omosa et al. (2021); Morkel and Sibanda (2022)	8
Goals	Chilisa (2015, 2024); Chilisa et al. (2016); Chilisa and Malunga (2012); Chilisa and Mertens (2021); Cloete (2016); Dlakavu et al. (2022); Fish (2022); Frehiwot, (2019, 2022); Gaotlhobogwe et al. (2018); Keney and Reid (2026); Blaser‐Mapitsa (2022); Masvaure and Motlanthe (2022); Mbava (2019); Mertens (2023a); Murgatroyd and Feront (2023); Omosa et al. (2021); Tirivanhu (2022)	18
Theoretical background	African Evaluation Association (2021); Boadu (2025); Chilisa (2015); Chilisa et al. (2016); Gaotlhobogwe et al. (2018); Chilisa and Malunga (2012); Chilisa and Mertens (2021); Frehiwot (2019); Kane and Archibald (2023); Montrosse‐Moorhead et al. (2024); Murgatroyd and Feront (2023); Omosa (2019); Schröter et al. (2025, 2026); Sibanda and Ofir (2021); Tirivanhu (2022); Uwizeyimana (2020).	17
Values & Ethics	African Evaluation Association (2021); Boadu (2022); Chilisa, (2015, 2024); Chilisa et al. (2025); Chilisa and Mertens (2021); Cloete (2016); Frehiwot (2022); Kane and Archibald (2023); Maikuri et al. (2022); Mapitsa & Ngwato, 2020); Montrosse‐Moorhead et al. (2024); Ouédraogo et al. (2025); Schröter et al. (2025, 2026); Sibanda et al. (2023)	15
Methodologies	African Evaluation Association (2021); Badjo (2022); Chilisa (2015); Chilisa et al. (2016); Chilisa et al., 2018; Chilisa & Malunga, 2012); Chilisa & Mertens, 2021); Dlakavu et al. (2022); Fish, 2022); Frehiwot, (2019, 2022); Maikuri et al. (2022); Blaser‐Mapitsa, 2022; Masvaure and Motlanthe (2022); Mazigo (2024); Mazigo, Mwaijande, et al. (2024); Mbava (2019); Mbava and Chapman (2020); Mertens (2023a); Murgatroyd and Feront (2023); Sefa‐Nyarko et al. (2024); Tirivanhu (2022, Watera (2025)	23
Practice	Abrahams (2025); Chapman et al. (2021); Chapman et al. (2026); Murgatroyd and Feront (2023)	4
Gaps and areas for further exploration	Chilisa (2015, 2024); Chirau et al. (2022); Cloete (2016); Dlakavu et al. (2022); Fish (2022); Frehiwot (2022); Kane and Archibald (2023); Mapitsa et al. (2019); Murgatroyd and Feront (2023); Ofir (2013); Ouédraogo et al. (2025); Tirivanhu (2022); Tirivanhu et al. (2018); Uwizeyimana (2020)	15
Future trajectory	Chapman et al. (2026); Chilisa, (2015, 2024); Chilisa and Mertens (2021); Chirau and Ramasobana (2022); Dlakavu et al. (2022); Frehiwot (2022); Keney and Reid (2026); Moilwa (2022); Morkel and Sibanda (2022); Murgatroyd and Feront (2023); Mutsikiwa and Mazongonda (2025); Ofir (2013); Omosa (2019); Ouédraogo et al. (2025); Pophiwa and Saidi (2022); Sibanda and Ofir (2021); Tirivanhu et al. (2018)	15

## RESULTS

In response to the study's guiding questions, we present a narrative synthesis of the 53 included papers that traces the evolution of MAE and reveals the paradigms, principles, values, and methods that underpin the framework. We also present three MAE case studies through which we identify parallels between MAE and community psychology praxis. We highlight how MAE's Indigenous orientation can enrich decolonial evaluation practices, advance epistemic justice, and support contextually grounded, culturally responsive community psychology practice. Finally, we outline gaps in MAE literature and future directions for advancing MAE and consider how integrating MAE can enhance the capacity and practice of community psychologists working in international and culturally diverse settings. We begin by outlining the evolution of MAE.

### The birth of MAE

The evaluation of development efforts in Africa was dominated by international aid agencies, large non‐governmental organizations (NGOs), and evaluators from the Global North until around the 1980s, when African scholars, researchers, activists, policy analysts, and evaluators began advocating for alternative perspectives on African development and its evaluation. By the late 1990s, African scholars' concerns coalesced into a broader critique of the dominance of Western‐centric evaluation paradigms and methods (e.g., results‐based management, log frames, experimental methods) and questioning of their relevance and implications for African development (Chilisa, 2024; Cloete, 2016; Ouédraogo et al., 2025). A key development towards an African‐centric approach to evaluation came through the establishment of the African Evaluation Association (AfrEA) in 1999 at AfrEA's inaugural conference in Nairobi, Kenya, in response to collective concerns that evaluation practice in Africa was donor‐driven and overlooked community priorities (Chilisa et al., 2025; Chilisa, 2015, 2024). AfrEA serves as the regional umbrella for Africa's evaluation practitioner community. It is composed of the national voluntary associations of professional evaluators from across the continent. AfrEA was established to promote African‐rooted and African‐led evaluations that focus on contributing to relevant, transformative, and sustainable development on the continent (African Evaluation Association, 2007; Chilisa, 2015). AfrEA advocates for African‐led and African‐owned evaluations that reflect Africa's indigenous knowledge, values, culture, and lived experiences (Chilisa, 2015, 2024; Frehiwot, 2019; Khumalo, 2022; Mbava & Chapman, 2020; Mbava, 2019; Ouédraogo et al., 2025; Sefa‐Nyarko et al., 2024).

Considering that evaluation capacity was still emerging in many parts of Africa at the time of AfrEA's foundation, and the field had limited prominence then, AfrEA aspired to establish evaluation as both a discipline and profession (Chilisa et al., 2025; Chilisa, 2024; Cloete, 2016; Ouédraogo et al., 2025). Its main strategies included strengthening evaluation capacity‐development, professionalization, knowledge‐sharing, and advocacy for program evaluation standards tailored to African contexts. The ongoing formation of national evaluation associations and the compilation of a comprehensive database of evaluators during this period enhanced networking and collaboration across the continent (Omosa, 2019).

The MAE framework itself was birthed in 2007, at the 4th AfrEA Conference in Niamey, Niger during a discussion session, titled “Making Evaluation Our Own,” (African Evaluation Association, 2007; Chilisa et al., 2025; Chilisa, 2024; Ofir, 2018). The discussion prompted the African evaluation community to assess the paradigms guiding evaluation practice across the continent critically and culminated in the vision for African‐rooted and African‐led evaluation. Emphasis was placed on recognizing how African values and worldviews could shape contextually grounded, relevant evaluation frameworks, and advance evaluation‐focused theoretical and practice scholarship within Africa (African Evaluation Association, 2007; Chilisa, 2024; Ofir, 2018).

Although the vision for African‐led evaluation initially gained traction, it lost momentum after the conference until it was deliberated on in great depth at an African Thought Leaders' Forum on Evaluation for Development during AfrEA's 2012 conference at the Bellagio Center in Italy. The forum culminated in the AfrEA Board's decision to adopt a formal strategy to develop and implement MAE as a guiding approach for evaluation in Africa (African Evaluation Association, 2013; Chilisa, 2015, 2024; Ofir, 2018). The decision to pursue MAE as a guiding framework led to significant developments, including the establishment of the *African Evaluation Journal* in 2013 and the commissioning of Professor Bagele Chilisa, a prominent African scholar, to develop a comprehensive synthesis publication that provided deeper insights into the MAE concept (Chilisa, 2015). Thereafter, more perspectives and publications emerged, expanding the discourse on MAE and elaborating its tenets (Ofir, 2018). Over the past decade, MAE has continued to gain traction, driven by the decolonizing knowledge agenda that emphasizes African‐grounded epistemologies and Indigenous knowledge systems (Chilisa, 2024; Masvaure & Motlanthe, 2022; Murgatroyd & Feront, 2023; Tirivanhu et al., 2018; Tirivanhu, 2022).

### Entangled roots

Although MAE is an African‐rooted and African‐led evaluation movement, some pioneering efforts towards its development were supported by international development agencies. Cloete (2016) notes that the United Nations Children's Fund (UNICEF) played a central role in establishing a network of evaluation practitioners in Africa through a 1977 Nairobi initiative that supported evaluation capacity development for UNICEF and other agencies across eight countries on the continent. UNICEF also sponsored the inaugural Pan‐African Conference of Evaluators in Nairobi‐ the meeting at which AfrEA was formed, attended by 300 participants from 26 African countries. The conference was spearheaded by Dr. Mahesh Patel, then working at UNICEF, who became AfrEA's first President (1999–2002). International agencies continued to influence MAE's development, for instance, the Norwegian Agency for Development Cooperation (NORAD). NORAD offered to fund an early test case for an African‐rooted evaluation approach at the African Evaluation Association, 2007 conference, while the Bill and Melinda Gates Foundation sponsored one of the first landmark synthesis studies on MAE in 2015 (Chilisa, 2015). Recently, UNICEF was engaged in AfrEA's 2025 African School of Evaluation, a flagship capacity‐strengthening initiative for the African evaluation community (African Evaluation Association, 2026). Furthermore, a recent major study (Chilisa, 2024) mapping the application of MAE in African evaluation projects was funded by EvalPartners, a global network collaboratively funded by United Nations agencies and other international development organizations, including regional evaluation networks.

Findings from this study indicate that these patterns of external funding and partnership have contributed to a perception among some African evaluation scholars that, despite its intellectual foundations being rooted in Africa, MAE is being orchestrated from outside Africa (Chilisa, 2024). Similar concerns were echoed in a recent empirical study by Mutsikiwa and Mazongonda (2025). These tensions and entanglements illustrate that MAE is emerging within a donor‐dependent evaluation ecosystem (Khumalo, 2022). This complex context influences how MAE is understood and received across the continent. Hence, clarifying what MAE is matters because the complexity of the ecosystem it exists within can obscure the nature of the approach and what values MAE stands for.

### What is MAE?

MAE is an African‐rooted evaluation framework that positions African values, cultures, worldviews, histories, and Indigenous knowledge systems as central to how evaluation is conceptualized and practiced (Badjo, 2022). It is defined as an evaluation that promotes African values, is guided by AfrEA standards, and uses contextually appropriate methods or approaches to align with the worldviews, lifestyle, and priorities of African people (Omosa et al., 2021; Omosa, 2019). Drawing on interviews with African evaluation scholars, Chilisa (2024, p. 11) expanded this definition, conceptualizing MAE more broadly as “an umbrella framework that includes evaluation approaches guided by the diverse philosophies, cultures, values, histories, languages, Indigenous and local knowledge systems, experiences, and practices of the African people, have a decolonization intent and apply the AfrEA principles to evaluation practice.” This expanded conceptualization builds on earlier definitions by emphasizing MAE's decolonial orientation while recognizing the diverse cultural, linguistic, historical, and knowledge contexts across the continent. This conceptualization positions MAE as a flexible framework that is responsive to the sociocultural contexts where evaluation takes place. As a movement, MAE resists the “blind borrowing of Western values and standards to evaluate programs in Africa” (Chilisa & Mertens, 2021, p. 245). MAE asserts that evaluation in Africa must emerge from the dynamics of daily community life, be grounded in African epistemologies, and guided by the priorities, lived experiences, and values of African communities (African Evaluation Association, 2021; Chilisa, 2015, 2024; Ouédraogo et al., 2025).

MAE is guided by nine core principles: relationality, responsibility, reverence, reciprocity, respectful representation, reflexivity, responsivity, adherence to community rights and regulations, and decoloniality (Chilisa & Mertens, 2021). Together, these principles articulate MAE's relational commitments as well as the ethical and epistemological orientations that distinguish it from conventional evaluation approaches. They guide evaluator engagement with communities and other interest‐holders. The principles are defined in Table 4.

**Table 4:** Summary of MAE principles.

Principle	Definition
Relationality	Prioritizes mutually respectful, long‐term relationships among evaluators, funders, and communities; acknowledging community perspectives as central to the evaluation process
Responsibility	Makes ethical, balanced judgments that center community interests
Reverence	Indigenous values, worldviews, spirituality, and ways of knowing are integral to understanding community perspectives
Reciprocity	Examines if intervention is meaningful, beneficial, and sustainable for the community, while recognizing their contributions and shared responsibility with funders and evaluators to sustain impact
Respectful representation	Enables meaningful community representation through equitable and accessible processes throughout the evaluation process
Reflexivity	Reflects on positionality and privilege to address power imbalances and ensure practices honor community values, wellbeing, and dignity
Responsivity	Emphasizes being alert and flexible, adapting to new insights or changes in context through continuous learning
Rights and regulations	Enables communities to enact agency via meaningful engagement throughout agenda setting, evaluation design, data collection, analysis, dissemination, and implementation
Decolonization	Develops evaluation frameworks with communities, drawing from their indigenous worldviews, values, and practices rather than imposing

*Note*: Adapted from Chilisa and Mertens (2021).

#### MAE purposes

MAE advocates for a shift in evaluation thinking and practice so that it is led and owned by African people, guided by African values and informed by African Indigenous knowledge, rather than relying predominantly on external or Western frameworks (Dlakavu et al., 2022; Keney & Reid, 2026; Mbava, 2019; Tirivanhu, 2022). It embraces African ways of being, seeing, knowing, and relating as essential to evaluation thinking and practice within the broader pursuit of transformative and lasting change in the continent (Chilisa, 2024; Murgatroyd & Feront, 2023; Ouédraogo et al., 2025). MAE's emergence as an alternative approach to evaluation seeks to bring a deeper understanding of the development interventions that are implemented in Africa through developing context‐specific, African‐centered evaluation strategies that embrace cultural, epistemic, and historical diversity (Keney & Reid, 2026; Masvaure & Motlanthe, 2022).

MAE also seeks to shift evaluation away from centering the donor towards accountability to communities and prioritizing community interests (Chilisa & Mertens, 2021; Chilisa, 2015, 2024; Mertens, 2023a; Murgatroyd & Feront, 2023; Ouédraogo et al., 2025). Being cognizant of historical context, MAE also aims to shift evaluation power dynamics through engaging in processes that empower African evaluators and communities (Frehiwot, 2019; Murgatroyd, 2023). This power shift is critical because African evaluators and communities, despite being the people most affected by development interventions, have historically been confined to the least influential aspects of evaluation, such as translation, data collection, and enumeration, while international evaluators retain control over the substantive decisions that guide evaluation design, and analysis. MAE seeks to reposition African evaluators and communities at the center of evaluation practice. Achieving this colossal shift requires changing the architecture of evaluation. Hence, MAE calls for the development and application of evaluation practices and methodologies drawing from African cultures, worldviews, knowledge systems, philosophies, and paradigms (Chilisa et al., 2016; Chilisa, 2024; Dlakavu et al., 2022; Fish, 2022; Frehiwot, 2022; Masvaure & Motlanthe, 2022; Murgatroyd & Feront, 2023).

#### Theoretical underpinnings

To examine how this reorientation unfolds, the following section outlines the paradigms, principles, values, and methods that underpin MAE. Table 5 synthesizes these MAE foundations and contrasts them with dominant Western, donor‐centric, development evaluation frameworks.

**Table 5:** Comparing MAE to dominant Western‐centered evaluation frameworks.

Dimension	MAE	Dominant Western donor‐centric frameworks
Purpose	Contributing to collective well‐being, transformative change and sustainability, restoration, and reparation of historical injustice by centering African priorities, values, and culture in evaluation thinking and practice	Monitoring efficiency, documenting results, demonstrating value for money, cost‐effectiveness, impact, guiding resource allocations, enforcing accountability for development results
Accountability flows	Emphasizes accountability to community members: those most impacted by the intervention	Emphasizes accountability to funders, commissioners, governments, and institutions responsible for resource allocation
Paradigm	Indigenous, relational, ecosystem‐focused, transformative	Positivist, post‐positivist, emphasizing objectivity,
Epistemology	Evaluative knowledge is relational, contextual, rooted in lived experience, and collaboratively produced with communities	Evaluative knowledge is objective, discoverable, measurable/quantifiable, generalizable, and produced by experts through systematic inquiry and analysis
Guiding Principles	Relationality, responsibility, reciprocity, reverence, respectful representation, reflexivity, responsivity, community rights, and decoloniality	Transactionality, cost‐effectiveness, procedural ethics, neutrality, compliance, checking boxes, procedural rigor, transparency, efficiency, compliance, and methodological consistency
Approach	Participatory, consultative, collaborative, community‐engaged, community‐led, bottom‐up, meaningful engagement with full range of interest holders across all phases of the evaluation process	Expert‐led, structured around predefined evaluation questions, indicators, and frameworks, top‐down, technocratic, minimal community engagement in decision making
Methods	Indigenous methods grounded in community ways of being and knowing, storytelling, dialogue, observation, collective reflection, proverbs, collective dialogue gatherings, arts‐based approaches, participatory inquiry, open to mixed methods where valuable	Standardized, emphasizing measurement., randomized‐controlled trial, surveys, indicators, experiments, quasi‐experiments, administrative data, and other methods
Processes	Affirming, accessible, flexible, iterative, generative, adaptive, responsive, relational, empowering, enabling mutual learning, reciprocity,	Often extractive, linear, time‐bound, standardized, and organized around predetermined designs, timelines, indicators
Prioritizing resources	Recognizes need to allocate sufficient time and resources for relationship and trust building, dialogue, and sustained community engagement as necessary investments for meaningful evaluation.	Provides limited time and minimal funding, prioritizing rapid data collection over relationship‐building; relies on short‐term, project‐bound engagement with little or no investment in sustained community connections.
Valuing	Values relationships, dignity, agency, lived experiences, Indigenous knowledge, community‐defined priorities, and transformative outcomes	Often privileges measurable/quantifiable impact, comparability
Community role	Knowledge holders, experts, co‐interpreters, and partners in meaning‐making	Often participants, informants, respondents, beneficiaries, and objects of data collection whose experiences are collected as evidence
Evaluator role	Relational actor, facilitator, co‐learner, collaborator, advocate, ally	Agent of funders/institutions, objective, detached, neutral expert, technical assessor, methodological and analytical authority
Dissemination	Findings shared through accessible language, media formats and processes that facilitate learning and support collective action, e.g., workshops, interactive presentations/gatherings, publications in community languages, artwork, audio/visual, or physical artifacts	Findings typically communicated through formal reports, presentations, publications, and decision‐making channels designed for funders or institutions. Communities excluded from dissemination

MAE rests on three theoretical foundations: the Ubuntu philosophy (Mangena, 2016; Tamale, 2020; Uwizeyimana, 2020), the Transformative framework (Mertens, 2009, 2023a, 2023b, 2025), and Indigenous paradigms (Chilisa et al., 2025; Chilisa et al., 2016; Chilisa, 2024; Gaotlhobogwe et al., 2018; LaFrance & Nichols, 2008; LaFrance et al., 2012). Each contributes distinct but complementary insights that define MAE's ethical, methodological, and epistemological stance. Ubuntu provides the moral and relational foundation; the Transformative framework contributes a social justice and social change orientation; and Indigenous paradigms situate evaluation within African worldviews and knowledge systems. Collectively, these foundations establish MAE as a relational, contextually grounded, and decolonial framework for evaluation.

##### Ubuntu philosophy

A widely recognized thread within value systems across diverse Bantu‐speaking cultures and regions in Southern, Eastern, and Central Africa is a commitment to collective personhood rooted in mutually dependent relationships and shared responsibility (Chigumadzi, 2023; Kane & Archibald, 2023; Omosa, 2019). This essence of dignity, reciprocity, and interconnectedness is summed up in the philosophy of Ubuntu, which is reflected in the moral precept, “umuntu ngumuntu ngabantu” which means a person is a person through other persons (Chilisa & Mertens, 2021; Mangena, 2016; Tamale, 2020). The philosophy resonates across African cultures and countries, where it is captured in various languages (Chilisa, 2024; Frehiwot, 2019; Mangena, 2016; Tamale, 2020; Uwizeyimana, 2020). Ubuntu is an aspirational concept for personal and communal behavior. A person who embodies Ubuntu expresses empathy, interest in others' points of view, hospitality, compassion, generosity, selflessness, respect, supportiveness, and cooperativeness (Graness & Kopf, 2024; Ngomane, 2019). Embodying these qualities is an active, conscious choice that requires effort, and, at times, sacrifice and ongoing reflection about being in relationship with others (Simba, 2026). Ubuntu remains a lived practice for many Africans today, particularly among rural communities where relational values and communal interdependence continue to shape daily life (Graness & Kopf, 2024). The relational values embodied in Ubuntu shape African ethical praxis and resonate with philosophies in other Indigenous societies, such as whānau and whanaungatanga, which emphasize kinship and the nurturing of relationships in Māori society (Sibanda & Ofir, 2021).

Critics have raised questions about the extent to which Ubuntu can serve as a representative philosophical foundation for African‐centered evaluation. They argue that while Ubuntu offers a compelling relational framework, it does not reflect the philosophical orientations of all African communities and is only one among many relational philosophies on the continent (Kane & Archibald, 2023; Uwizeyimana, 2020). Counterarguments emphasize that Ubuntu should not be understood as a singular or universal African philosophy, but rather as a broadly shared worldview of relational existence common across many African societies that aligns closely with MAE's vision of evaluation grounded in community values (Chilisa, 2024).

Ubuntu guides MAE's centering of relational accountability within evaluation processes (Schröter et al., 2025, 2026). This perspective reimagines the evaluator not as a neutral, detached observer, but as a relational actor whose accountability is grounded by a shared commitment to advocate for the well‐being of the community (Chilisa & Mertens, 2021; Norins et al., 2023). Similar relationally centered concepts characterize community psychology (Trickett, 2009) and CRE approaches such as Indigenous Evaluation and CRE (Cram et al., 2018; Hood et al., 2015; Hopson, 2009).

Ubuntu is unique in its emphasis on communal interdependence and its focus on how one lives well in community with others. As a reflection of the embracing of MAE by African evaluation professionals, Ubuntu is now evident in the ethical guidelines for evaluation practice in Africa promulgated by its leading voluntary professional associations (Sibanda et al., 2023). This shift is significant because Ubuntu challenges the relational dynamics embedded in broader development aid and its evaluation. Historically, development interventions have unfolded through interactions where funders are positioned as owners of resources, holders of knowledge, and bearers of the development mandate engaging with communities framed as recipients of resources and objects of inquiry rather than as partners with knowledge, agency, resources, and aspirations, and with whom a shared vision of development could be imagined and realized. This arrangement is transactional: the donor provides particular types of resources, and the community is expected to receive and account for them (Chilisa et al., 2025). Evaluation then focuses on demonstrating that resources have been used as intended by the donor rather than jointly examining whether shared aspirations are being realized.

This contrasts sharply with an Ubuntu‐informed relational lens, which understands aspirations, resources, efforts, and outcomes as shared and embedded within relationships and approaches development evaluation as a collaborative, relational process. Instead of “seeing” communities and engaging in this kind of relationship, dominant, donor‐centric evaluation undermines community sovereignty, dignity, and agency (Sibanda & Ofir, 2021). In response, Ubuntu embodies a restorative justice orientation. It demands the repair of historical harm where African communities have been denied the recognition of their full humanness, dignity, agency, and the legitimacy of their ways of being and knowing (Maikuri et al., 2022; Malherbe et al., 2025; Morkel et al., 2026). These “injured relations” (Chigumadzi, 2023) are restored through transforming the harmful engagement into a mutually respectful relationship grounded in dignity, agency, shared responsibility, and reciprocity. This kind of restorative justice is both reactive to relational injury and proactive as an ethical guideline for navigating the relationship (Davis, 2019).

By emphasizing relational accountability and epistemic justice, MAE reimagines ethical integrity in evaluation as a living expression of Ubuntu principles and values (Mapitsa & Ngwato, 2020).

MAE's ethical framework also derives from the African Evaluation Principles, which were developed by AfrEA between 2019 and 2021. These principles were introduced in response to the growing demand for African‐centric evaluation, replacing previous versions such as the African Evaluation Guidelines that had shaped the field for over 20 years but were rooted in American evaluation standards (African Evaluation Association, 2021; Patel, 2002, 2013). As MAE gained momentum, AfrEA initiated a broad consultative process (2019–2021) to revise and replace their guidelines to reflect evaluation that is “Made in Africa,” where African people understand the purpose of evaluation and shape evaluation processes to be responsive to their context (Chilisa, 2015).

In this spirit, the current African Evaluation Principles center Ubuntu by defining ethical evaluation practices as grounded in integrity, respect, fairness, and accountability to communities (African Evaluation Association, 2021). This calls for evaluation processes that enable community agency to set priorities and lean on their ways of knowing (Chilisa, 2024). The principles promote contextually relevant, inclusive evaluation processes that center community expertise and reflect a commitment to collective well‐being, consistent with the Ubuntu philosophy (African Evaluation Association, 2021).

##### Merten's transformative research and evaluation framework

Although it was developed in the U. S. context, Mertens' (2009) Transformative framework resonates among evaluators in the African context because it centers social justice, human rights, and the voices of marginalized communities in evaluation processes. The framework was introduced in the U.S. in response to concerns about the misrepresentation and exclusion of marginalized groups, such as persons with disabilities and racialized minority groups, in research and evaluation (Mertens & Cram, 2016; Mertens, 2023a, 2023b). Using a transformative lens in evaluation involves acknowledging the complex power relationships embedded in the historical, sociocultural, and political context that contribute to inequality, and being responsive to those dynamics through engaging the full range of social actors in the evaluation process and ensuring the perspectives of the most marginalized are prioritized (Mertens & Cram, 2016). Because the framework is oriented toward social transformation and challenging the status quo for the benefit of society's most marginalized members, it emphasizes marginalized community members' perspectives, understandings, and experiences to inform the evaluation and interpret results (Mertens & Wilson, 2018; Mertens, 2023b; Miller & Mertens, in press). Among U.S. evaluation theories, the Transformative framework closely reflects community psychology's values and orientation to action and social change (Miller, 2017).

Within the context of MAE, the Transformative framework places African communities' transformative aims at the heart of the evaluation process. As we previously noted, development evaluations in Africa typically prioritize the perspectives of and questions posed by donors. Navigating the complex terrain of evaluation in Africa requires evaluators to engage critically with the power dynamics that shape the broader evaluation ecosystem (Mapitsa & Ngwato, 2020; Murgatroyd & Feront, 2023). The Transformative framework is explicit in its mandate to interrogate and counter these dynamics. Centering community perspectives, as the Transformative framework requires, marks an ethical and axiological shift toward social and epistemic justice, in which the power to frame problems, envision transformative change, and shape evaluation processes rests with those most directly impacted by the context, affirming their lived experiences, and honoring their ways of knowing (Abma et al., 2020; Chilisa & Mertens, 2021).

##### Indigenous paradigms

In evaluation practice, decisions on methodologies and methods, processes, priorities, participants, and dissemination of findings are guided by paradigms (Chilisa & Mertens, 2021). The evaluation garden, also called the Garden of Evaluation Approaches—a visual framework that guides practice through illustrating different evaluation approaches in a structured “garden” metaphor, positions MAE as rooted in, or reflective of, an Indigenous paradigm (Montrosse‐Moorhead et al., 2024; Schröter et al., 2025). This positioning acknowledges that MAE draws from a broader intellectual tradition of Indigenous paradigms undergirded by a holistic perspective that honors relationships among people and between humanity and nature (Chilisa, 2020; LaFrance & Nichols, 2008; Sibanda & Ofir, 2021). In these paradigms, the meaning of merit and worth is derived from community members' lived experiences of programs and policies rather than from pre‐determined funder‐defined indicators. Methods that reflect the traditional ways in which communities learn are favored (Chilisa, 2024; LaFrance et al., 2012; Mazigo, 2025; Schröter et al., 2025; Sibanda & Ofir, 2021).

There is no singular Indigenous paradigm because the diversity of Indigenous evaluation frameworks reflects distinct cultural contexts and post‐colonial experiences. For example, Māori traditions emphasize relational principles such as whanaungatanga (relationships) and kaitiakitanga (guardianship), which are deeply embedded in Māori cosmology and social practice (Smith, 1999). These differ from the foundational values of Joan LaFrance's Indigenous evaluation framework, which prioritizes valuing community and family, honoring a sense of place, recognizing individual strengths, and respecting sovereignty (Mark et al., 2025). LaFrance's framework underscores the importance of meaningful engagement and respecting everyone and the unique contributions they bring to an evaluation. In her view, evaluation must be an affirming rather than a diminishing process. Bowman (2020) Indigenous framework emphasizes place‐based identity and honoring the legal, cultural, and political sovereignty of Indigenous communities. Her recent work grounds practice in the Lunappe seven directions (Bowman, 2025), and emphasizes community ownership of evaluations, including data and copyright over all knowledge products produced via evaluation processes (Bowman, 2025; Bowman Lunaape/Mohican & Bremner Métis, 2024). While echoing LaFrance's principles, Bowman's work is distinct in its focus on governance and intergovernmental relationships. Together, these frameworks illustrate that Indigenous evaluation is a constellation of approaches in which Indigenous rights, values, sovereignty, and cultural practices inform all aspects of an evaluation, including who owns it.

In Africa, Indigenous paradigms push back against the continued dominance of Western evaluation approaches and methods, as well as favor the leadership of African evaluators. African Indigenous paradigms respond to the epistemic erasure that persists in so far as African communities and African evaluators and their values, priorities, ways of being and knowing are absent from or sidelined in development evaluations (Chilisa, 2015, 2024; Chilisa et al., 2016; Chilisa & Mertens, 2021; Mertens, 2024; Murgatroyd & Feront, 2023). A distinctly African Indigenous paradigm for evaluation calls for a fundamental reconceptualization of evaluation epistemology and methodology that draws on African Indigenous knowledge (Chilisa, 2015, 2024; Chilisa & Mertens, 2021; Fish, 2022; Mertens, 2023a; Uwizeyimana, 2020). This reconceptualization recognizes Indigenous knowledge as a fully legitimate scientific system grounded in relational ways of knowing and on equal footing with Westernized approaches to science (Badjo, 2022; Chilisa, 2020, 2024; Chilisa et al., 2016; Gaotlhobogwe et al., 2018; LaFrance et al., 2012). In practice, this means tapping into the knowledge, customs, and practices embedded in community life to define intervention success and gather evidence of program performance rather than impose a predetermined framework that may be culturally inappropriate. In the next section, we explore the methodologies that emerge from an African Indigenous paradigm.

### Methodologies

MAE favors methodologies that are rooted in relational epistemologies, which call for the use of qualitative, participatory, dialogic, data‐collection methods such as storytelling, which aid understanding of context and align evaluation processes with the daily life of communities (African Evaluation Association, 2021; Badjo, 2022; Chilisa, 2015, 2020; Chilisa & Mertens, 2021; Frehiwot, 2019; Mazigo, 2025; Mbava, 2019; Mbava & Chapman, 2020; Mertens & Wilson, 2018; Murgatroyd & Feront, 2023; Tirivanhu, 2022). Storytelling holds deep historical significance in Africa since it is a foundational medium for societies that preserve and transmit knowledge orally through kinship‐based cultural institutions (Chilisa, 2015; Omosa et al., 2021; Tuhiwai Smith, 1999). MAE data collection methods typically reflect the ordinary ways in which people relate and learn through storytelling, proverbs, and observation (Dlakavu et al., 2022). Within these traditions, proverbs are one evaluative tool to distill Indigenous knowledge into practical wisdom and offer guidance for collective reflection (Badjo, 2022; Mazigo, 2024, 2025; Mazigo, Mwaijande, et al., 2024). Proverbs such as *Kinyozi hajinyoi* (“A barber does not shave himself”) and *Nyani haoni kundule* (“The ape does not see its own backside”) emphasize collaboration, recognizing that understanding the value and limitations of an intervention requires engagement with diverse viewpoints.

MAE also draws on open community dialogue forums such as Baraza, Inkundla, and Lekgotla that reflect how communities collectively engage in deliberative processes to resolve conflict, solve problems, or seek consensus in decision making (Chilisa, 2020, 2024; Chilisa et al., 2016; Chilisa et al., 2025; Gaotlhobogwe et al., 2018; Malherbe et al., 2025; Mbava & Chapman, 2020; Watera, 2025). Across the continent, variations of collective dialogue formats have long served as foundational platforms for the exchange of ideas and perspectives, reflecting historical reliance on participatory, relational processes to guide collective action. Collective dialogues remain vital spaces for public discourse to ensure accountability for community development initiatives, cultivate ownership, and build trust among communities, evaluators, and other development actors (Chilisa et al., 2016; Maikuri et al., 2022; Mbava & Chapman, 2020; Watera, 2025).

Culturally grounded, community‐led evaluative practices are not new innovations but longstanding African traditions that contemporary approaches to evaluation, such as MAE, seek to reclaim and strengthen (Badjo, 2022). This reclamation embodies the principle of Sankofa. Originating from Twi, a widely spoken Akan language in Ghana, Sankofa means “go back and gather.” Symbolized by a bird turning its head backward to retrieve an egg, Sankofa signifies drawing wisdom from the past to guide the future. The Sankofa bird is a widely recognized symbol representing an ongoing movement among Africans to reconnect with cultural heritage to address present and future challenges (Preston, 2025).

### Case studies

Our scoping review identified few published case studies of evaluations that use the MAE approach. MAE scholars have also commented on the fact that there are a limited number of case examples illustrating MAE practice, which limits the framework's ongoing refinement (Chilisa, 2015, 2024; Fish, 2022; Murgatroyd & Feront, 2023; Tirivanhu, 2022). We briefly present the three MAE cases we identified through our searches.

#### Case 1: South Africa's national monitoring and evaluation system

Abrahams (2025) reviewed the development of South Africa's national monitoring and evaluation (M&E) system as a compelling case study that reflects how African indigenous evaluation systems can develop organically in response to local sociopolitical conditions, while still engaging with global evaluation thinking and practice. Abrahams describes how South Africa's trajectory from apartheid to a democratic nation grappling with the enduring structural inequalities created a unique evaluation ecosystem rooted in restorative governance priorities. The evaluation system is oriented toward addressing historical injustices, improving service delivery for previously disadvantaged populations, and strengthening public accountability through mechanisms that require government institutions to demonstrate that their policies are effective in restoring equity. Abrahams' critical reflection illustrates how evaluation in African contexts is shaped by restorative and transformative priorities such as redressing historical injustices, which positions evaluation as a tool for social transformation rather than a purely technical exercise, as often emphasized in Western traditions. His analysis echoes Chilisa and Mertens' (2021, p. 245) view of MAE as resisting the uncritical application of Western values and standards in Africa.

#### Case 2: Youth development in KwaZulu‐Natal

Murgatroyd and Feront (2023) offer a rare and detailed account of MAE principles in practice within development programming. Their case makes visible the restorative and transformative commitments highlighted by Abrahams (2025). Their case examines the process of integrating MAE principles and Ubuntu philosophical tenets into the evaluation of two youth development programs. The programs were implemented by a non‐government organization (NGO) operating in the eco‐tourism sector in partnership with local corporate donors in Northern KwaZulu‐Natal, South Africa. The programs address youth unemployment and economic marginalization across five rural communities by tailoring support to university graduates aged 18–27 who are seeking work experience, and youth aged 18–35 without tertiary training who are engaged in the informal economy and in need of entrepreneurship and business support.

In line with MAE's emphasis on relational epistemologies and ethical engagement informed by Ubuntu philosophy, the evaluators prioritized building relationships with NGO staff, youth, and other interest holders. This relational commitment was reflected in the lead evaluator's 2‐month immersion in the NGO's activities prior to the evaluation. The immersion period culminated in an engaged process of co‐creating the evaluation framework with a diverse set of interest holders comprising program participants, tribal leaders, NGO staff, and donor representatives.

The evaluators used participatory and dialogic methods, which are hallmarks of MAE, to engage the community in collective reflections on their experiences of the program. Through this process, the evaluators surfaced tensions between donor and community values. The evaluators illustrate how shifting from donor‐driven quantitative metrics focused on outputs, such as income generation and business growth, to a set of community‐defined relational indicators of success, such as family well‐being, social standing, and community upliftment through service provision, created a more expansive understandings of the program's value. This shift in what was valued and who held the power to define value embodied the transformative ethos of MAE, where program value is framed through community worldviews rather than funder priorities. If success were defined solely by the donor‐determined indicator, the evaluation would have failed to capture the nuances of salient relational and gendered values, such as increased capacity to nurture families, accumulate household assets, and serve the community. The evaluators played a significant role in broadening the funder's understanding of the community context and community values. By centering community perspectives, the evaluators enriched the evaluation in ways that reflect MAE's contextually grounded, culturally responsive, transformative mandate and embody its grounding in Ubuntu philosophy.

#### Case 3: Training evaluators

Extending MAE's transformative agenda into evaluator education, Chapman et al. (2021) applied MAE through a pedagogical lens, focusing on a “Made in Africa” approach to Africa‐rooted evaluator training and curriculum design in South Africa. The program evaluation master's program they teach was established in 2008 to convey global advances (largely Western) in evaluation theory to emerging African evaluators. However, in 2015–2016, the sociopolitical landscape shifted dramatically with the emergence of the #RhodesMustFall and #FeesMustFall student protest movements in South Africa. These movements challenged the dominance of Western ideas in African universities, rejecting the idea of African universities as mere pipelines for Western knowledge. They called for decolonization of curricula and institutional culture and demanded decommodification of higher education. The movements catalyzed an ideological shift that demanded urgent curricula change to recognize African people as creators of global knowledge rather than objects of study or passive consumers of western knowledge.

These student movements occurred in parallel to the development of MAE. In response, Chapman and colleagues revised the program evaluation introductory course to reflect MAE principles and tenets. They prioritized African evaluation scholars' texts and case studies, incorporated an evaluation client as a co‐learner in the classroom, creating an experiential model that enabled students to engage with real‐world evaluation challenges in the African context and to practice context‐grounded, participatory, collaborative approaches that are central to MAE. Chapman et al. designed a course that facilitated critical engagement with the limitations of conventional Western textbooks when applied in complex evaluations scenarios in Africa.

The revised curriculum provided a transformative learning process in which the instructors, students, and evaluation client arrived at a mutual understanding of the program's vision. They co‐determined the evaluation questions and co‐created the evaluation framework as MAE recommends. What emerged was a relational, organic, shared form of learning about evaluation that mirrors MAE's commitment to empowering, context‐grounded evaluation practice embedded in daily life experiences and community meaning‐making. A recent collaborative follow‐up study with graduates of the course found that decolonial, MAE‐oriented training prompted graduates to question the dominance of Western theories and be more attentive to contextual complexity, especially when choosing designs and methods to adequately capture intervention impact in ways that prioritize community interests (Chapman et al., 2026).

## DISCUSSION

This scoping review synthesized literature on the history, tenets, and practice of MAE. The review aimed to take stock of what has been written about MAE, identify gaps in the emergent MAE literature, and assess the suitability of MAE for use by community psychologists. MAE grew from resistance to the colonial dynamics embedded in development aid and its evaluation. Like other Indigenous evaluation approaches, MAE responds to the need for CRE approaches that are relevant to the sociocultural and political contexts in which development programs operate. MAE is grounded in an ecosystemic, relational approach to evaluation that is anchored in Indigenous paradigms and a social justice orientation. MAE elevates African communities' priorities and their aims for social transformation in place of the priorities and aims of non‐Africans. Its restorative justice orientation stems from the Ubuntu relational philosophy. MAE reclaims African ways of being and knowing through its call for community participatory processes and use of dialogic methods that mirror ordinary community life and decision‐making.

### Key parallels between community psychology and MAE

It is evident from our scoping review that MAE and community psychology share multiple values and commitments. These include a commitment to community‐driven, contextualized, practice, and inquiry. Both call on inquiry processes that attend closely to community's sociopolitical histories, values, and structural power dynamics that bear on priority social problems (Abrahams, 2025; Dazzo, 2026; Dazzo et al., 2023; Mapitsa & Ngwato, 2020; Reyes Cruz & Sonn, 2011; Trimble et al., 2012). MAE's emphasis on relational accountability and systemic interdependence (Chilisa & Mertens, 2021; Sibanda & Ofir, 2021) is conceptually similar to Kelly's ecological metaphor (Kelly, 2006), which reimagines communities as dynamic ecosystems shaped by sociocultural norms, resource flows, and historical context. The ecological perspective and social justice frameworks that guide community psychology resonate with MAE's ecosystemic, participatory, and transformative approaches to evaluation.

Community psychology and MAE also overlap substantially in their emphasis on epistemic justice practices in which communities engage as co‐creators of knowledge rather than passive subjects (Mertens, 2024; Perkins et al., 2023; Prilleltensky, 2003, 2008; Reich et al., 2017; Sonn, 2016; Wallerstein & Duran, 2011). Both MAE and community psychology prioritize partnerships with historically disenfranchised communities to work collaboratively in ways that actively support community agency and self‐defined interests (Jason et al., 2019; Mertens, 2024; Miller, 2014; Minkler & Wallerstein, 2003; Morris, 2015; Sonn, 2016; Springett & Wallerstein, 2011; Tirivanhu, 2022). Moreover, both position evaluators as facilitators of collaborative engagement among diverse evaluation ecosystem constituencies; the purpose of gathering evaluative evidence is to serve as a catalyst for critical conversations around varied interest holders' values and priorities (Chapman et al., 2026; Jason et al., 2019; Kelly, 2006; Miller, 2014; Murgatroyd & Feront, 2023; Perkins et al., 2023; Reich et al., 2017). As community psychologists' intervention development, implementation, and evaluation processes are rooted in a social justice orientation which centers community priorities, interests and values, community psychologists are well‐positioned to use frameworks like MAE to challenge the top‐down approaches that are traditionally used in development evaluation.

To advocates of decolonial evaluation strategies, many evaluations continue to fall short of being equitable, culturally responsive, and justice‐oriented (Symonette et al., 2020). Evaluation aimed at supporting transformation “remains a niche practice and a major leap from how evaluation is most often undertaken” (Buckton et al., 2025, p.5). Hence, as the evaluation field explores more context‐specific, culturally responsive practice emerging from and aligned to the diverse epistemologies, histories, and worldviews of communities, there are opportunities for community psychologists to bring their unique disciplinary values and skills to bear. Community psychologists operating in Africa or other international and multicultural settings that are similar in history and culture can engage with Indigenous evaluation approaches like MAE and contribute to its ongoing development. Given the small number of available case studies that document the use of MAE, community psychologists working in Africa and similar settings are uniquely positioned to contribute to filling this gap.

MAE and its grounding in Ubuntu philosophy calls attention to the possibility that inquiry processes like evaluation can reproduce rather than redress inequality. Ongoing critical reflection on how evaluation processes may reproduce inequality and how they might evolve towards more socially just futures is crucial (Symonette et al., 2020). Reflective practice enables examination of positionality and privilege to actively identify and address power imbalances. With its emphasis on interconnectedness, mutual respect, and cooperation (Mangena, 2016; Ngomane, 2019), Ubuntu offers a powerful lens to support this type of reflection. As a lived practice governing daily interactions and with a uniquely African sensibility about how to live well in the world with others, Ubuntu can be applied to examine the ways in which community psychologists working in African contexts engage in interactions and practices that honor community values, wellbeing, and dignity. This kind of reflective practice positions evaluation using the MAE approach as a dynamic, imaginative process of engagement with people and values to recognize what is morally significant in each evaluation context, consistent with Schwandt's view of evaluation as a moral activity (Schwandt, 2024). Reflection on moral reasoning could foster deeper awareness, humility, and accountability, enabling community psychologists to align evaluation efforts closely with African values and understandings of justice, equity, and collective wellbeing.

Such reflection is significant because evaluation is fundamentally a process of valuing. Judgments about the merit, worth, or significance of an intervention are never value‐neutral; they reflect the priorities and worldviews of those involved (Lincoln & Guba, 2013; Schröter et al., 2025; Schwandt, 2015, 2024; Schwandt & Gates, 2021). MAE offers practical strategies for community psychologists to move away from imposing expert judgments of intervention success and lean on community‐defined perspectives rooted in African values, relational worldviews, and indigenous knowledge (Bhatia et al., 2025; Murgatroyd & Feront, 2023; Readsura Decolonial Editorial Collective, 2022; Readsura Decolonial Editorial Collective & Malherbe et al., 2023).

### Advancing the MAE framework

As an emerging and evolving framework, MAE continues to be refined through ongoing scholarly engagement. MAE is gaining traction but the lack of clear and tested practice guidance or exemplar cases illustrating how to implement MAE in program evaluation practice limits its use (Chilisa, 2015, 2024; Fish, 2022; Murgatroyd & Feront, 2023; Ouédraogo et al., 2025; Tirivanhu, 2022). MAE raises important questions about how evaluation grounded in African values can contribute to transformative change across the continent. Empirical research documenting MAE processes and outcomes emerging from its community‐led, participatory, and narrative‐driven methods is necessary to advance the approach beyond discourse (Fish, 2022). Expanding research on MAE and its applications into a substantial body of practice‐based knowledge through the documentation and dissemination of exemplar cases can support learning, critical engagement, and broader integration of MAE within the development evaluation sector (Dlakavu et al., 2022). MAE is not yet widely embraced by African development actors such as governments, foundations, and those that commission evaluations, which may reflect both its limited application and the limited research devoted to its development (Chilisa, 2024). However, more research on MAE and its use in practice may help establish MAE as a viable alternative to common evaluation approaches used in the context of assessing development aid in Africa. The limitations of donor‐centric evaluation frameworks continue to become increasingly evident in Africa, while African‐rooted approaches such as MAE may become important for advancing sustainable development across the continent (Rahim et al., 2025). Community psychologists working in Africa have a unique potential role to play in MAE's ongoing refinement, given the commonalities in our theories and orientations to inquiry and action.

### Limitations

Our scoping review of the literature on MAE is not without its limitations. By focusing exclusively on English‐language publications, we excluded scholarship available in other colonial languages that are common to the continent, notably French and Portuguese, as well as publications that may be available in tribal languages. Including MAE scholarship and experiences from Francophone and Lusophone country contexts may have offered valuable perspectives on MAE's foundations and emergent principles in African contexts that are shaped by French, Belgian, and Portuguese colonization. In addition, it is likely that there is a gray literature in the form of confidential evaluation reports submitted to donors from which much could be learned on early experiences using MAE to guide evaluations in Africa. Future research on MAE could seek to examine the practice experiences of those evaluators who practice MAE but who have not published their experiences.

## CONCLUSION

This scoping review explored the tenets underpinning MAE and examined how its integration can enhance the capacity and practice of community psychologists working in Africa and similar cultural contexts. The synergy between MAE and community psychology reflects immense potential to both enrich and draw from one another to advance MAE as a tool for social justice and community‐driven transformative change. MAE offers community psychology a decolonial, contextually grounded approach to evaluation that centers African worldviews, values, and Indigenous knowledges. MAE also provides a powerful lens for ongoing reflection through its roots in Ubuntu philosophy, enabling community psychologists to critically assess whether their practices honor the dignity of African people, strengthen their agency, and contribute to meaningful transformative change in Africa.

Community psychologists' expertise in community‐based and collaborative forms of action research, theoretical grounding in concepts such as empowerment (e.g., Christens, 2012) and the ecological metaphor (e.g., Kelly, 2006), and experience in pursuit of systems change all position community psychologists to contribute to MAE's continued development and growing empirical evidence base on its practice. Rooted in African epistemologies, MAE aligns with critical and decolonial approaches that challenge the marginalization of Indigenous knowledge and community agency in evaluation and research. Its relevance, therefore, extends beyond Africa to any context where dominant evaluation frameworks fail to adequately reflect local knowledge, values, and priorities, and to community psychologists who are committed to the aims of decolonial inquiry.

## CONFLICT OF INTEREST STATEMENT

The authors declare no conflicts of interest.

## Data Availability

Data sharing is not applicable to this article as no datasets were generated or analyzed during the current study.
